# Crosstalk between the mTOR and DNA Damage Response Pathways in Fission Yeast

**DOI:** 10.3390/cells10020305

**Published:** 2021-02-02

**Authors:** John-Patrick Alao, Luc Legon, Charalampos Rallis

**Affiliations:** 1 ZEAB Therapeutic, University of East London, Stratford Campus, Water Lane, Stratford, London E15 4LZ, UK; john.p.alao@zeabtherapeutic.com; 2 School of Health, Sport and Bioscience, University of East London, Stratford Campus, Water Lane, Stratford, London E15 4LZ, UK; l.legon1601@uel.ac.uk; 3 School of Life Sciences, University of Essex, Wivenhoe Park, Colchester CO4 3SQ, UK

**Keywords:** TORC1, TORC2, nutrients, caffeine, rapamycin, Rad3

## Abstract

Cells have developed response systems to constantly monitor environmental changes and accordingly adjust growth, differentiation, and cellular stress programs. The evolutionarily conserved, nutrient-responsive, mechanistic target of rapamycin signaling (mTOR) pathway coordinates basic anabolic and catabolic cellular processes such as gene transcription, protein translation, autophagy, and metabolism, and is directly implicated in cellular and organismal aging as well as age-related diseases. mTOR mediates these processes in response to a broad range of inputs such as oxygen, amino acids, hormones, and energy levels, as well as stresses, including DNA damage. Here, we briefly summarize data relating to the interplays of the mTOR pathway with DNA damage response pathways in fission yeast, a favorite model in cell biology, and how these interactions shape cell decisions, growth, and cell-cycle progression. We, especially, comment on the roles of caffeine-mediated DNA-damage override. Understanding the biology of nutrient response, DNA damage and related pharmacological treatments can lead to the design of interventions towards improved cellular and organismal fitness, health, and survival.

## 1. The Mechanistic Target of Rapamycin Pathway in Cell Growth and Metabolism

The mechanistic target of rapamycin (mTOR) is a phosphatidylinositol 3-kinase-related serine/threonine kinase central to a signaling network that regulates cellular metabolism in eukaryotic cells in response to various extracellular and intracellular stimuli, including nutrient and hormone signals, stress, and growth factors [1,2]. The kinase mTOR is also implicated in cellular health span through modulation of nutrient-sensitive pathways [3]. Originally identified in yeast mutants [4], mTOR is highly conserved and has now been identified in all eukaryotic cells including flies, nematodes, and mammalian cells [5,6,7,8,9]. The fission yeast *Schizosaccharomyces pombe* has been widely used as a model to study eukaryotic molecular mechanisms, including the conserved mTOR pathway. *S. pombe* contain homologues for Rheb, a GTP-binding protein, and an upstream activator of mTOR [10], as well as the TSC1–TSC2 complex that negatively regulates Rheb [11,12,13].

**Figure 1:**
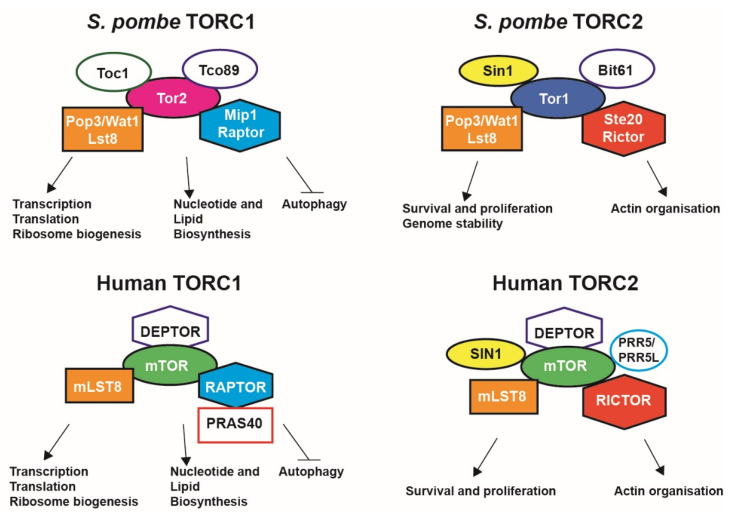
*Schizosaccharomyces pombe* and human TORC1 and TORC2 complexes. Each complex is composed by a TOR kinase (predominantly Tor2 for TORC1 and Tor1 for TORC2 fission yeast complexes) and at least four partners identified through mass spectrometry approaches. Lst8 is a common partner for both TORC1 and TORC2. However, in fission yeast, Mip1 (RAPTOR), Tco89, and Toc1 are associated with TORC1 only, while Ste20 (RICTOR), Sin1, and Bit61 are associated with TORC2. In human complexes, DEPTOR is found in both complexes, while PRAS40 is associated with TORC1 and PRR5/PRR5L with TORC2. Each complex has pivotal roles in metabolism, cell organization, growth, and survival (see main text for details). mTOR, mechanistic target of rapamycin.

In most eukaryotes, mTOR forms the central component of multiprotein complexes termed TORCs [14]. *S. pombe* contains two mTOR homologues, Tor1 and Tor2, and two TORC complexes, TORC1 and TORC2 (Figure 1). The TOR kinases form the catalytic subunit of the complexes, with the rapamycin sensitive TORC1 containing Tor2, while TORC2 contains Tor1. Both complexes are composed of several other proteins and are structurally and functionally distinct [15,16,17]. There are slight differences between the structure of the TORC complexes in *S. pombe* and the TORCs found in mammalian cells (Figure 1). While both complexes are still present, mammalian cells only contain one form of the TOR gene (MTOR). The mTOR protein comprises the catalytic subunit of both mTORC1 and mTORC2; the complexes also contain orthologues as well as different proteins to those found in *S. pombe* [16,18,19]. 

TORC1, unlike its counterpart, is inhibited by the macrolide compound rapamycin, which forms a complex with its intracellular receptor FKBP12. Inhibition of mTOR with rapamycin mimics nitrogen starvation in fission yeast. For strong inhibition of TORC1 in *S. pombe*, rapamycin needs to be accompanied by caffeine, which also inhibits the complex [20,21,22,23]. TORC1 is regulated upstream by numerous intracellular and extracellular stimuli, including in mammalian cells, the cell cycle regulator p53 upon DNA damage from chemical agents. In contrast to yeast, mTORC1 also activates p53 under pro-growth conditions [24]. The TORC1 complex regulates ribosomal biogenesis and protein synthesis primarily through the phosphorylation of two distinct downstream effectors, S6K activated by phosphorylation and eIF4E binding proteins (4EBP), which are inactivated upon phosphorylation [25,26]. The complex also has implications over other anabolic processes including lipid and nucleotide synthesis. Furthermore, the complex also regulates several catabolic processes including autophagy and protein degradation to balance the metabolic state of the cell [1] (Figure 1). 

**Figure 2:**
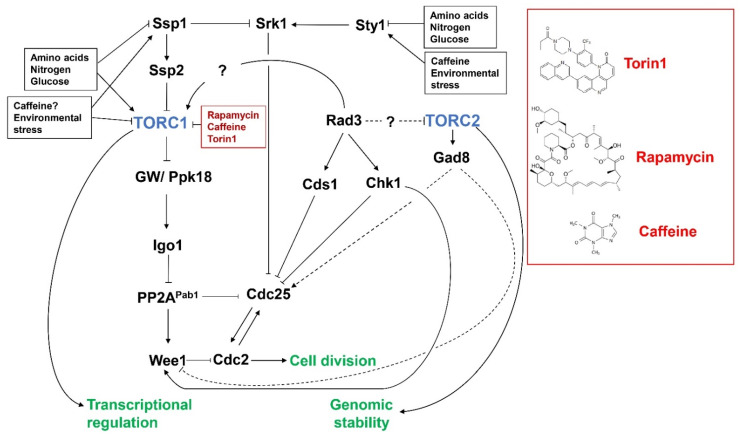
Interaction of DNA damage response (DDR), environmental stress response (ESR), and TORC1/TORC2 signaling in *S. pombe*. TORC1 and TORC2 play distinct roles in regulating cell growth and division as well as responses to environmental stresses and nutrient availability. Activation of Rad3 by DNA damage or replication stress leads to the activation of Chk1 and Cds1, respectively. Chk1 and Cds1 inhibit Cdc25 and activate Wee1 (or Mik1) to inhibit Cdc2 and delay cell cycle progression. Direct inhibition drives cells into mitosis via the Ppk18-Igo1-PP2A^Pab1^ pathway in the presence of DNA damage, suggesting Rad3 may maintain TORC1 activity during cell cycle arrest. Conversely, TORC2 is required for the resumption of cell cycle progression once DNA repair has been affected. Rad3 may also regulate TORC2 activity during the DDR. Caffeine may directly inhibit TORC1 as a low affinity ATP competitor. Alternatively, caffeine may indirectly inhibit TORC1 via activation of the ESR and Ssp1-Ssp2 (AMPK) pathways.

TORC2 is not inhibited by rapamycin, owing to the structure preventing the binding of the rapamycin/FKBP12 complex [27]. Torin1 is a known ATP competitive inhibitor of mTOR and blocks the catalytic active site, preventing downstream phosphorylation in both complexes [28]. Because of the absence of a specific TORC2 inhibitor, the complex is less studied and more remains to be elucidated when compared with its counterpart [29]. TORC2 in fission yeast is not essential for cellular proliferation, however, it is essential for sexual differentiation and entry into the stationary phase, which are two of the main starvation responses in fission yeast [17,30]. Interestingly, TORC2 has also been shown to work in tandem with TORC1 to regulate the phosphorylation of S6K in *S. pombe* [31]. Alongside this, the TORC2 complex is also responsible for actin dynamics, telomere integrity, and cell size [32,33,34,35]. TORC2-deficient *S.pombe* are sensitive to several stress conditions including osmotic and oxidative stress and, importantly, DNA damage and replicative stress [36]. Furthermore, the complex has also been implicated in the maintenance of the genome in *S. pombe*, elucidating a further connection between the TORC2 complex and DNA damage response pathways [37] (Figure 1 and Figure 2).

## 2. DNA Damage Response (DDR) Signaling: Cdc25, Wee1, and DNA Damage CheckPoint Activation

Cells continuously suffer various forms of DNA damage from both endogenous and exogenous sources. To avoid the “fixing” of mutations and/or chromosome mis-segregation during mitosis, cells must arrest cell cycle progression and delay mitosis (DNA damage checkpoints) until damaged DNA has been repaired [38]. While various forms of DNA damage (ranging from base misincorporation and additions to double strand breaks) occur, in *S. pombe*, Rad3 is the major regulator of the DNA damage response and a member of the PIKK family [39,40,41,42,43,44] (Figure 2). Rad3 is a homologue of the mammalian ataxia telangiectasia mutated (ATM) and ataxia telangiectasia and Rad3 related (ATR) and *Saccharomyces cerevisiae* (*S. cerevisiae*) Mec1 proteins [42,43].

The timing of mitosis is regulated by the opposing actions of the Cdc25 phosphatase and Wee1 kinase on Cdc2 tyrosine 15 phosphorylation, and hence activity [45,46]. DNA damage checkpoint activation and maintenance, thus, require the dual regulation of Cdc25 and Mik1/ Wee1 activity to effectively inhibit Cdc2 [47]. Additionally, cells must be able to effectively resume cell cycle progression once DNA repair has been completed, linking DNA damage checkpoint activation and resumption of cell division [44,48]. In general, these pathways are evolutionarily conserved and follow a general pattern of DNA damage detection, signal activation, signal amplification/transmission, and execution. The Rad3 kinase and its homologues are recruited to sites of DNA damage, leading to their activation via autophosphorylation [40,49]. This facilitates the recruitment of adaptor proteins such as Crb2, which allow the cell cycle phase specific recruitment and activation of the downstream kinases Cds1 and Chk1 during the S- and G2-phases of the cell cycle, respectively [50,51,52].

During the S-phase, deleterious events such as nucleotide depletion stalled replication forks and DNA strand breaks result in the Rad3-dependent hyper-phosphorylation and activation of Cds1. Cds1 in turn directly inhibits Cdc25 by phosphorylating inhibitory serine/threonine residues, while inducing the accumulation of the Wee1-related kinase Mik1. The S-phase specific Mik1 kinase regulates Cdc2 tyrosine 15 phosphorylation in a Rad3-dependent manner [53,54,55]. Alternatively, DNA damage sustained during G2 results in Chk1 and Wee1 activation [53]. Chk1 phosphorylates Cdc25, leading to its export from the nucleus and Rad24 (a 14-3-3 family type protein)-dependent sequestration and accumulation within the cytoplasm [56,57,58]. The nuclear export of Cdc25 is, however, not required for its inhibition, suggesting direct inhibition by Cds1 and Chk1 [58,59]. Additionally, studies using Cdc25 mutants lacking Cds1/Chk1 phosphorylation sites suggest the existence of a redundant nuclear ubiquitin-dependent degradation pathway [60,61,62]. The cytoplasmic accumulation observed in *S. pombe* following cell cycle arrest may thus facilitate the rapid resumption of cell cycle progression following the completion of DNA repair [48,63]. Wee1 also accumulates in response to DNA damage and is itself a target of activating Chk1 phosphorylation. The dual inhibition of Cdc25 phosphatase activity and increased Wee1 activity thus results in Cdc2 tyrosine 15 phosphorylation and inhibition by a “double- lock” mechanism [47,64].

*S. pombe* cells also transiently arrest cell cycle progression and then rapidly progress through mitosis in response to nitrogen deprivation and various environmental stresses [65,66,67]. The environmental stress response (ESR) pathway is regulated by the Sty1 kinase (Figure 2), a homologue of the human p38MAPK kinase [68,69,70]. Sty1 activation results in srk1 induction and accumulation of the Srk1 kinase. Srk1 in turn phosphorylates Cdc25, leading to its sequestration and accumulation within the cytoplasm [65]. Interestingly, the transient activity of Srk1 appears to be dependent on the CaMKK homologue Ssp1. *S. pombe* mutants lacking *ssp1* become elongated and arrest cell cycle progression in G2, following exposure to heat stress, osmotic stress, and glucose deprivation [71,72,73,74]. Srk1 levels are elevated in *ssp1Δ* mutants and the deletion of *rad24* or *srk1* is sufficient to restore cell cycle progression under stress conditions [73,74]. Ssp1 also activates the AMPK homologue Ssp2, which in turn inhibits TORC1 signaling [75] (Figure 2). Ssp1 may thus integrate Sty1 signalling with TORC1 activity to facilitate the resumption of cell cycle progression following exposure to environmental stresses.

## 3. TORC1, Caffeine and the DNA Damage Response Pathway

Given the links between the Sty1 environmental stress response (ESR) and Rad3 DNA damage response (DDR) regulated pathways, it has been proposed that these pathways may also interact with TORC1 and TORC2 signaling [24,76,77,78,79]. For instance, glucose deprivation, which inhibits TORC1 signaling, induces the accumulation of Cds1 [80]. Cds1 normally accumulates during S-phase or activation of the replication and S-phase checkpoints [50,55].

Under nitrogen-rich conditions, active TORC1 inhibits the Greatwall kinase homologue Ppk18, which prevents its activation of the endosulphine Igo1 [45,81]. Following transfer to a poor nitrogen source, Ssp2 mediated TORC1 inhibition activates Ppk18 and Igo1, leading to the inhibition of the PP2A phosphatase catalytic subunit Pab1 [33,82,83]. Under normal cell cycle conditions, PP2A^Pab1^ delays the timing of mitosis by negatively regulating Cdc25 and positively regulating Wee1 activity [67,84]. TORC1 inhibition following nitrogen deprivation or chemical inhibition advances cells into mitosis, in a manner dependent on Sty1 and Ppk18-Igo1-PP2A^Pab1^ signaling, resulting in Cdc2 activation [67,83,84]. Furthermore, pab1Δ mutants exhibit a “wee” phenotype (small-sized cells upon mitosis), confirming its role as a regulator of mitotic progression [85]. TORC1 thus indirectly delays cells’ cycle progression via the negative and positive regulation of Cdc25 and Wee1, respectively [67]. As Rad3 indirectly inhibits Cdc2 via Cdc25 inhibition and Wee1 activation, these findings suggest TORC1 activity re-enforces DNA damage checkpoint signaling by maintaining PP2A^Pab1^ activity [62,78] (Figure 2).

Caffeine is a methylxanthine and among the most widely consumed neuroactive substances in the world [86]. Early studies clearly indicated that caffeine overrides DNA damage signaling in both yeast and mammalian cells [87,88,89,90]. Rad3 and its homologues were initially reported to be inhibited by caffeine in vitro and in vivo [88,90]. These findings have proved controversial, as caffeine inhibits several members of the PIKK family. In addition, caffeine induced ATM hyperactivation and failed to inhibit the phosphorylation of its downstream targets [91]. In *S. pombe*, Rad3 and the Rad51/Rad54 regulated DNA pathways are required for tolerance to caffeine, suggesting it might induce low level DNA damage [92]. Caffeine also failed to inhibit Cds1 and Chk1 phosphorylation in *S. pombe*, in contrast to the deletion of *rad3* [62]. In mammalian cells, inhibition of PP2A activity inhibits DNA repair and ATM contributes to the activation of the phosphatase [38]. Furthermore, PP2A and related phosphatases negatively regulate the DDR and are required for eventual progression towards mitosis [93]. In this regard, the ability of caffeine to override DNA damage signaling has generated much interest, but the precise underlying mechanisms remain unclear [62,78,94].

More recent studies suggest that the TORC1 complex is the target of caffeine in both yeast and mammalian cell lines [21,22,95] (Figure 2). This raises the intriguing question of if and how caffeine might override DNA damage checkpoint signaling by inhibiting TORC1 activity. The effect of caffeine on cell cycle progression in *S. pombe* mimics that of nitrogen deprivation, TORC1 inhibition, and other environmental stresses [62,67,76]. Indeed, inhibition of TORC1 activity with Torin1 advanced *S. pombe* cells into mitosis in a manner similar to nitrogen deprivation. This activity was associated with changes in the activity and/or expression of Cdc25 and Wee1 in both *S. pombe* and mammalian cells [67,76]. TORC1 inhibition results in the activation of Ppk18, which then activates Igo1, resulting in the inhibition of the PP2A^Pab1^ phosphatase, and increases and decreases in Cdc25 and Wee1 activity, respectively [45,81]. TORC1 activity would thus be required to enforce Rad3-mediated DNA damage checkpoint signaling to prevent the premature activation of Cdc2. Conversely, inappropriate TORC1 inhibition under genotoxic conditions would advance cells into mitosis with unrepaired DNA, leading to a loss of viability. This is indeed the case, as both caffeine and torin1 enhance DNA damage sensitivity in *S. pombe* [96]. Furthermore, caffeine appears to indirectly regulate the activity of both Cdc25 and Wee1. Thus, caffeine mediated-TORC1 inhibition might be sufficient to override DNA damage checkpoint signaling [62,77,78]. Under environmental stress conditions, cells undergo a temporary Sty1-mediated cell cycle arrest through Srk1-dependent Cdc25 inhibition. The cells are subsequently advanced into mitosis regardless of cell length [65]. These stresses also inhibit TORC1 signaling via the Ssp1 and Ssp2/AMP activated protein kinase (AMPK) pathways. *S. pombe* mutants lacking *ssp1* fail to accelerate mitosis following exposure to various environmental stresses and become greatly elongated [71]. It has been demonstrated that *ssp1Δ* mutants express elevated levels of Srk1, suggesting enhanced Cdc25 inhibition and an inability to resume cell division. Indeed, the deletion of *ssp1* is synthetically lethal in a *cdc25-22* strain background [71,74]. Furthermore, both *ssp1Δ* and *ssp2Δ* mutants fail to advance mitosis under low nitrogen conditions [75]. The Ssp2-mediated inhibition of TORC1 is thus required for the downstream activation of Ppk18 and advancement into mitosis under environmental and nutrient stress conditions [82].

Previous studies in *Saccharomyces cerevisiae* (*S. cerevisiae*) have identified TORC1 (Tor1) mutants that exhibit increased tolerance to caffeine. These studies suggested that, unlike rapamycin and other analogues, caffeine directly binds to Tor1 and acts as a competitive inhibitor of ATP [95]. It remains unclear if caffeine indirectly inhibits TORC1 signaling via the Ssp2/AMPK kinase pathway. Because the Sty1-regulated ESR pathway is required for tolerance to caffeine, it is possible that the drug also indirectly inhibits TORC1 signaling in *S. pombe* (Figure 2). It has also been noted that the inhibition of TORC1 by Torin1 differs from that of caffeine and rapamycin [62,67,97]. It also remains unclear if TORC1 regulates the timing of mitosis under normal cell cycle conditions by inhibiting Ppk18 until the unset of mitosis, or if PP2A^Pab1^ is negatively regulated by additional pathways [82]. Studies in *S. pombe* have demonstrated that Cdc25 and Wee1 are degraded at mitosis independently of currently identified phosphorylation pathways [84]. Further studies will be needed to dissect the precise effects of caffeine on TORC1 signaling in relation to cell division [62,78]. Despite its pleotropic effects on cellular physiology, caffeine may provide useful insights into the regulation of TORC1 and its downstream targets. Such studies could also lead to the development of therapeutic strategies to combat aging and increase the sensitivity of cancer cells to chemo- and radiotherapy.

## 4. Crosstalk between TORC2 and DNA Damage Response Pathways

In contrast to TORC1, the Tor1 containing TORC2 complex has been clearly implicated in mediating resistance to osmotic and nutritional stress responses as well as genotoxic agents in *S. pombe* [79]. Loss of Tor1 activity induced sensitivity to several genotoxic agents such as hydroxyurea (HU), Methyl methanesulfonate (MMS), UV, and camptothecin. Similarly, mutants lacking Gad8, a downstream target of Tor1, also exhibit sensitivity to genotoxic agents [32,37]. Recent studies implicating *gad8* mutants show that osmotic and nutritional stress responses appear to form a separate branch from genotoxic stress responses downstream of TORC2-Gad8 [98].

Interestingly, *tor1Δ* mutants were checkpoint proficient, but failed to resume cell cycle progression in a manner like wild type cells. Tor1 is thus not required for Rad3 mediated DNA damage checkpoint activation. In fact, deletion of *tor1* suppressed the short- term HU sensitivity of *rad3* and *cds1* mutants. Rather, *tor1Δ* mutants failed to dephosphorylate Cdc2 and failed to resume cell division [32]. Tor1 thus appears to be required for the reactivation of Cdc2 following exposure to HU and other genotoxic agents. In fact, Tor1 appears to promote mitotic progression as loss of *tor1* resulted in a slight increase in average cell length. Rad3 may simultaneously inhibit TORC2 activity until DNA damage repair has been completed [32,37]. 

More recent findings report Chk1 activation in *S. pombe* mutants that lack certain components of the TORC1 protein complex. *S. pombe* mutants lacking the TORC1 and TORC2 complex component Wat1/Pop3 sustain DNA damage through the effects of reactive oxygen species (ROS). This results in constitutive Chk1 activation and enhanced inhibition of Cdc25 and Wee1 [99,100]. Wat1/Pop3 is a component of both complexes and is required for the oxidative stress response. Loss of Wat1/Pop3 may thus induce DNA damage, because of ROS concentrations. Additionally, *wat1/pop3* mutants are unable to downregulate Chk1 activation in a manner like *tor1* mutants. These findings provide further evidence for a TORC2-dependent role in maintaining genomic stability [32,99,100].

## 5. Prospects for mTOR as a Chemo- and Radio-Sensitisation Therapeutic Target

Caffeine initially aroused much interest owing to its ability to sensitize cancer cells to the lethal effects of genotoxic agents [86]. Clinically, this activity is important as it would increase the therapeutic window of these agents and lower the side effects of chemo- and radio-sensitization [101,102,103]. More recently, caffeine has been shown to extend chronological lifespan (CLS) in a wide range of organisms. Furthermore, caffeine has been shown to have protective effects and against cancer and can improve clinical outcomes [104]. It is now apparent that caffeine exerts its effects on cell division via TORC1 rather than Rad3/ATM inhibition, and that these effects are mimicked by selective TORC1 inhibitors such as rapamycin and Torin1 [21,22,95]. Torin1 affected Cdc25 and Wee1 stability and cell cycle progression in both *S. pombe* and mammalian cell lines [67]. 

These findings suggest that TORC1 may serve as a therapeutic target for enhancing the chemo- and radiosensitivity of cancer cells. Further studies on caffeine-mediated DNA checkpoint override in the context of TORC1 and mTOR inhibition are thus highly desirable. Importantly, it remains unclear if caffeine functions as a direct inhibitor of TORC1 or partially mediates its effects via the activation of the AMPK pathway [71,75]. Such studies will be important as the role of AMPK in tumors is context-dependent [105,106,107]. Dissecting DNA damage checkpoint override by caffeine and other TORC1 inhibitors is likely to provide a stimulating and revealing field of study in the years to come.

## Data Availability

No new data were created or analyzed in this study. Data sharing is not applicable to this article.
